# Emotional distress and burnout at a fever clinic in China: Comparison between different periods of COVID-19

**DOI:** 10.3389/fpsyt.2023.1138361

**Published:** 2023-03-13

**Authors:** Wenqi Geng, Jinya Cao, Xia Hong, Jing Jiang, Jiaojiao Hu, Yanping Duan, Jing Wei

**Affiliations:** Department of Psychological Medicine, Peking Union Medical College Hospital, Chinese Academy of Medical Sciences and Peking Union Medical College, Beijing, China

**Keywords:** COVID-19, burnout, healthcare workers, China, anxiety, depression

## Abstract

**Background:**

Frontline healthcare workers (FHWs) experienced psychological stress and heavy workload during COVID-19 pandemic. This study examined the psychological symptoms and occupational burnout of FHWs in a fever clinic during different periods of the pandemic.

**Methods:**

A cross-sectional survey of FHWs in the fever clinic of a tertiary hospital was carried out during both the outbreak period and regular period of COVID-19. Psychological measurement instruments including Generalized Anxiety Disorder 7-item, the 9-Question Patient Health Questionnaire, the Maslach Burnout Inventory-Human Service Survey, and the General Self-Efficacy Scale were used to evaluate anxiety, depression, burnout, and self-efficacy, respectively. The correlation between clinical variables was explored.

**Results:**

A total of 162 participants were involved in this study, including 118 FHWs during the outbreak period (Group 1) and 44 FHWs during the regular period (Group 2). Anxiety symptoms were more prevalent in Group 2 (*x*^2^ = 27.477) while depressive symptoms were significantly more prevalent in Group 1 (*x*^2^ = 69.538). Burnout rate was higher in Group 2 (*x*^2^ = 29.526). Self-efficacy was higher in Group 1 (*t* = 3.194). Burnout was positively correlated with anxiety symptoms (*r*^2^ = 0.424) and negatively correlated with self-efficacy (*r*^2^ = −0.312).

**Conclusion:**

Anxiety, depressive symptoms and burnout were prevalent in FHWs during different periods of the COVID-19 pandemic. There is a tendency to be less depressed, but more anxious and burned out over time, although the severity of the pandemic is decreasing. Self-efficacy may be an important factor in protecting FHWs from occupational burnout. Support and intervention plans for FHWs should be made at the institutional level.

## 1. Introduction

Since December 2019, the COVID-19 pandemic has spread rapidly in China and around the world, becoming a public health emergency of global concern (1). Especially in the early days, the COVID-19 pandemic has put enormous pressure on governments and people around the world. The general public has had to cope with acute stress due to the uncertain source of disease, rapid transmission, and complexity of treatment (2). To date, the COVID-19 pandemic remains a major global public health issue and continues to pose a threat to all of humanity (3). Despite the increasing rate of vaccination against the virus, problems such as virus mutation, virus transmission and increased infection capacity remain prominent.

Every individual affected by the epidemic is facing great mental stress. WHO has identified addressing mental health needs as an essential part of the response to the COVID-19 pandemic (4), such as addressing public emotional reactions and stress among health workers. Medical staff were confronted with a variety of psychological stresses, including the risk of infection, high-intensity work stress, frustration at the lack of effective treatment, and loneliness in isolation (5). In the early days of the COVID-19 pandemic in China, a study of healthcare workers (HWs) in Wuhan (6) found that 50.4% of HWs exposed to the pandemic had clinically significant depressive symptoms. In another study of HWs in Beijing (7), 12.2% were depressed. Other studies around the world have found that during the first wave of the pandemic, HWs were under great mental stress and their mental health was significantly affected. In a study in Ethiopia (8), 58, 16.3, and 30.7% of HWs experienced moderate or severe stress, depression, and anxiety symptoms during the pandemic, and HWs’ poor coping was related to these psychological impairments, suggesting the need for psychological intervention for HWs. A study in Switzerland (9) found that 70% of HWs reported significant emotional stress and increased anxiety during the first wave of the pandemic, with a lack of protective equipment being an important source of stress. In the severely affected areas, the number of patients increased rapidly, far exceeding normal workload, and there was usually a serious shortage of personnel and supplies. The imbalance between resources and needs was first felt by frontline healthcare workers (FHWs). As the group most exposed to the disease, FHWs had a higher prevalence of anxiety, depression, and stress-related symptoms than the general public (10). Previous reviews of the psychological status of medical personnel in infectious disease outbreaks also found consistent evidence that gender, nurse occupation, and frontline working status are clear risk factors for psychological stress (11, 12), suggesting that psychological support for medical personnel needs to pay more attention to the female frontline nurse population.

Professional burnout was first described in 1975 by Freudenberger (13) on staff in a free medical clinic. Characteristics of occupational burnout in the context of physical and behavioral symptoms include increased anger, frustration, excessive rigidity and inflexibility in practice, and the appearance of depression characteristics. Those who are prone to burnout are often dedicated and committed to their profession. Burnout is not an acute condition but rather a chronic culmination of the effects of unsolvable, long-term work stress, professional responsibilities and the work environment. The three dimensions of burnout syndrome are emotional exhaustion, depersonalization or cynicism, and a decreased sense of personal accomplishment (13–15). Research indicates that burnout in healthcare professionals can lead to negative attitudes toward day-to-day work and a reduced focus on patients, which hinders medical safety and quality, and has serious consequences for the worker’s personal life (16–18). Professional burnout of FHWs during the COVID-19 pandemic has been reported in some studies. A study reported high rates of insomnia, burnout, and functional impairment among healthcare providers in Jordan during the first year of the COVID-19 pandemic (19). A study during the pandemic in Japan (20) found that more than 40% of nurses and more than 30% of radiological technicians and pharmacists met the criteria for occupational burnout. A study in Belgium (21) found that nearly half of HWs working on the front lines in the first wave of COVID-19 had significant occupational exhaustion, 28.8% had moderate or higher depression, 41.8% had moderate anxiety or higher, and 25.1% had moderate or higher stress, with increased workload and perceived support associated with these adverse outcomes. Researchers from Korea found that burnout had a direct effect on depression, anxiety, and physical and mental health in HWs (22). Combined, these factors can also pose a significant risk to the quality of patient management.

By 2023, many countries, including China, have adopted regular control measures to reduce the impact of the pandemic on the general public. However, FHWs were inevitably constantly faced with COVID-19. Recently, with more relaxed control measures adopted in China, the number of COVID-19 cases is expected to increase, possibly leading to more work-related stress in FHWs. To date, there have been few studies examining the current psychosomatic health status of FHWs during the regular period of the pandemic. In this study, we examine the psychological symptoms and occupational burnout of FHWs in a fever clinic during both the outbreak period and the regular period, in order to provide more evidence and help improve the psychosomatic intervention of FHWs during the pandemic.

## 2. Materials and methods

### 2.1. Study design and participants

**Figure 1:**
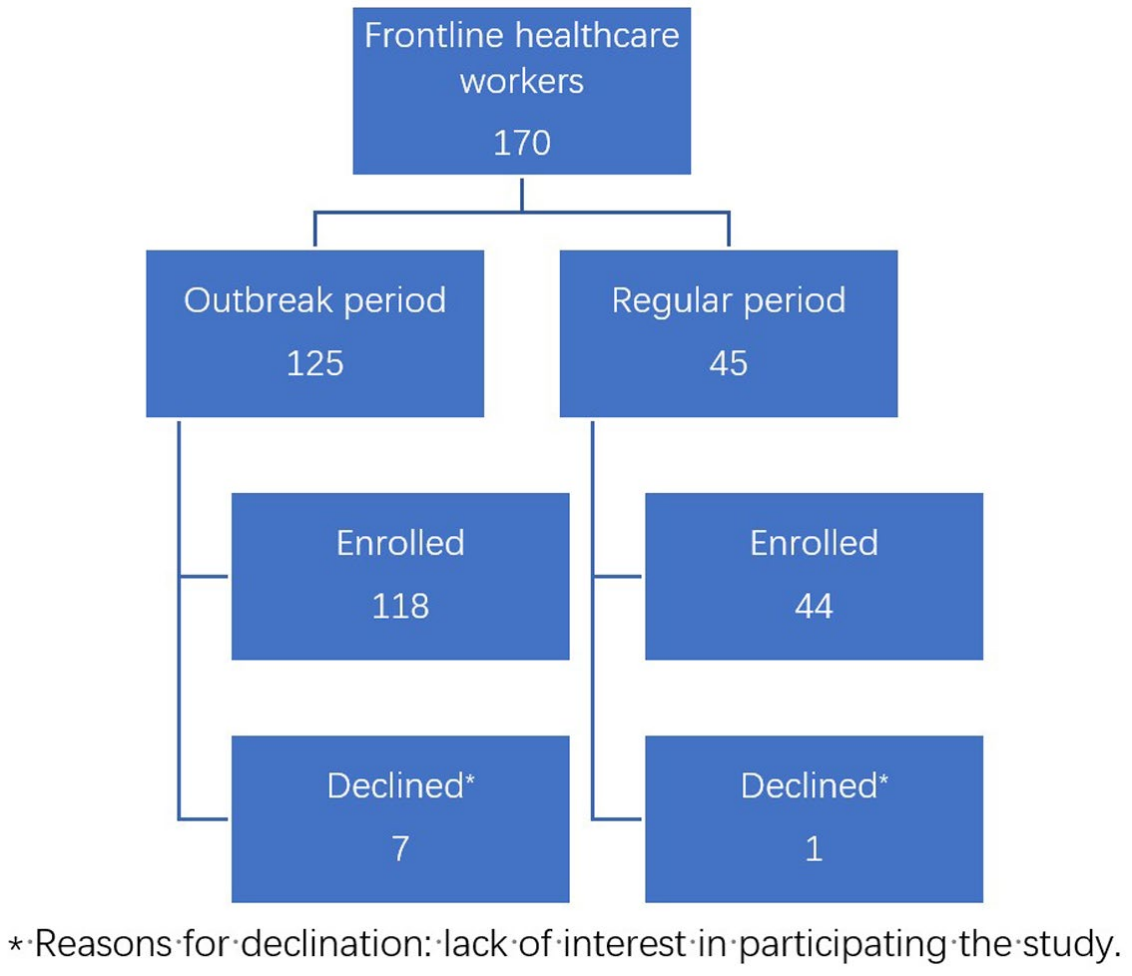
Flowchart of study subjects.

This study was designed as a single-center cross-sectional study. All FHWs working in the fever clinic of a tertiary hospital in Beijing during the designated time period were considered eligible for this study, which included the COVID-19 outbreak period (January 2020 to April 2020, group 1) and the regular period (October 2021 to November 2021). Two researchers (JJ and HJ) contacted FHWs by telephone and invited them to participate. Of the 170 FHWs invited, 162 agreed to join the study. The enrollment of participants is shown in Figure 1. Participants were divided into two groups based on when they worked in the fever clinic, and there was no overlap in participants. The survey was conducted by telephone during the COVID-19 outbreak (Group 1) or *via* an online questionnaire during the regular period (Group 2). Demographic and psychological data were collected.

### 2.2. Ethical considerations

The study was reviewed and approved by the ethics committee of Peking Union Medical College Hospital (approval number S-K1045), which is located in Beijing, China. Oral informed consent was obtained from each participant.

### 2.3. Psychological measurement instruments

Chinese validated versions of the following questionnaires were used to evaluate participants’ psychological symptoms: Generalized Anxiety Disorder 7-item (GAD-7) (23), Patient Health Questionnaire 9-item (PHQ-9) (24), Maslach Burnout Inventory-Human Service Survey (MBI-HSS) (25, 26), and General Self-Efficacy Scale (GSES) (27, 28).

GAD-7 consists of seven questions that assess the frequency of anxiety symptoms. Each question is scored from 0 (not at all) to 3 (almost every day), giving a total score of 0 to 21. Anxiety symptoms are defined as a GAD-7 score ≥ 5. A total score of 5–9, 10–14, and ≥ 15 are considered mild, moderate, and severe anxiety symptoms, respectively.

PHQ-9 consists of nine questions assessing the frequency of depressive symptoms. Each question is scored from 0 (not at all) to 3 (almost every day), summing up to a total score of 0 to 27. Depressive symptoms are defined as a PHQ-9 score ≥ 5. A total score of 5–9, 10–14, and ≥ 15 are considered mild, moderate, and severe depressive symptoms, respectively.

MBI-HSS is a 22-item instrument covering three aspects of burnout, emotional exhaustion (EE), depersonalization (DP), and personal accomplishment (PA). Each item has a 7-point Likert scale from “never” or 0 to “daily” or 6. We defined a 27 or higher EE score, a 10 or higher DP score, or a 33 or lower PA score as burnout for participants.

GSES is a 10-item self-rating scale that assesses the strength of an individual’s belief in his or her own ability to respond to novel or difficult situations and to cope with any associated obstacles or setbacks. For each item, there are four response choices from ‘not at all true’, which scores 1, to ‘exact true’, which scores 4. The scores for each of the 10 items are summed up to give a total score. The higher the score, the greater the individual’s generalized sense of self-efficacy.

### 2.4. Statistical analyses

All statistical analyses were performed using IBM SPSS Statistics 21.0.0.0. (IBM Corp., Armonk, NY, United States). Quantitative variables are described as mean ± standard deviation or median (interquartile range [IQR]) based on the normality of the variable. Categorical variables were described as frequencies (percentages). The Student’s t-test was used to compare the two groups for continuous variables. The Chi-square test was used to compare the distributions of categorical variables among the groups. The correlation between clinical variables was tested using Spearman’s correlation test. A value of *p* < 0.05 was considered statistically significant. This study was designed to search for clinical associations; therefore, only exploratory analyses are presented.

## 3. Results

A total of 162 participants completed the questionnaire, including 60 (37.0%) doctors, 92 (56.8%) nurses and 10 (6.2%) laboratory or radiology technicians (Table 1). The majority (71.0%) were female. Participants had an average age of 31.1 ± 6.5 years. Group 1 (G1) consisted of 118 FHWs who worked in the fever clinic during the outbreak period, while Group 2 (G2) contained 44 FHWs during the regular period. There were no significant differences in age, sex, and occupation (physician, nurse, or technician) between G1 and G2.

**Table 1:** Demographic information, psychological symptoms, burnout, and self-efficacy scores of participants.

	Group 1 (118)	Group 2 (44)	x^2^/t	*p*
Age	31.47 ± 6.60	30.18 ± 6.02		
Sex				
Male	32 (27.1%)	15 (34.1%)		
Female	86 (72.9%)	29 (65.9%)		
Occupation				
Doctor	48 (40.7%)	12 (27.3%)		
Nurse	61 (51.7%)	31 (70.4%)		
Technician	9 (7.6%)	1 (2.3%)		
Psychological symptoms				
Anxiety symptoms (GAD-7 ≥ 5)	13 (11.0%)	21 (47.7%)	27.477	<0.001
Depressive symptoms (PHQ-9 ≥ 5)	115 (97.5%)	19 (43.2%)	69.538	<0.001
Burnout	29 (24.6%)	32 (72.7%)	29.526	<0.001
EE	5 (4.2%)	19 (43.2%)	36.734	<0.001
DP	12 (10.2%)	24 (54.5%)	34.577	<0.001
PA	18 (15.3%)	21 (47.7%)	17.151	<0.001
Self-efficacy	2.93 ± 0.54	2.60 ± 0.64	3.194	0.002

GAD-7, The Generalized Anxiety Disorder 7-item; PHQ-9, The 9-Question Patient Health Questionnaire; EE, emotional exhaustion; DP, depersonalization; PA, personal accomplishment.

Anxiety symptoms were more prevalent in G2 (11.0% vs. 47.7%, *x*^2^ = 27.477, *p* < 0.001) while depressive symptoms were significantly more prevalent in G1 (97.5% vs. 43.2%, *x*^2^ = 69.538, *p* < 0.001). In G1, the mean score of GAD-7 was 0 (IQR 0–2). Nine (7.6%) participants had mild anxiety symptoms (GAD-7 score 5–9) and four (3.4%) had moderate symptoms (GAD-7 score 10–14). G2 had a mean score of 4.5 (IQR 2–9) in GAD-7. There were 16 (36.4%) participants in G2 with mild anxiety symptoms and 5 (11.4%) participants with moderate anxiety symptoms. None of the participants in either group had severe anxiety symptoms (GAD-7 score > 15). With depressive symptoms, G1 and G2 had mean PHQ-9 scores of 9 (IQR 7–10) and 4 (IQR 1–7). In G1, the number of participants with mild (PHQ-9 score 5–9), moderate (PHQ-9 score 10–14), and severe (PHQ-9 score ≥ 15) depressive symptoms were 84 (71.2%), 25 (21.2%), and 6 (5.1%) respectively. In G2, 15 (34.1%) participants had mild depressive symptoms, 2 (4.5%) had moderate symptoms, and another 2 (4.5%) had severe symptoms.

Occupational burnout rate was significantly higher in G2 than in G1 (24.6% vs. 72.7%, *x*^2^ = 29.526, *p* < 0.001). The three factors of burnout, emotional exhaustion (4.2% vs. 43.2%), depersonalization (10.2% vs. 54.5%), and personal accomplishment (15.3% vs. 47.7%) all reflected a similar trend. Self-efficacy was significantly lower in G2 than in G1 (2.93 ± 0.54 vs. 2.60 ± 0.64, *t* = 3.194).

**Figure 2:**
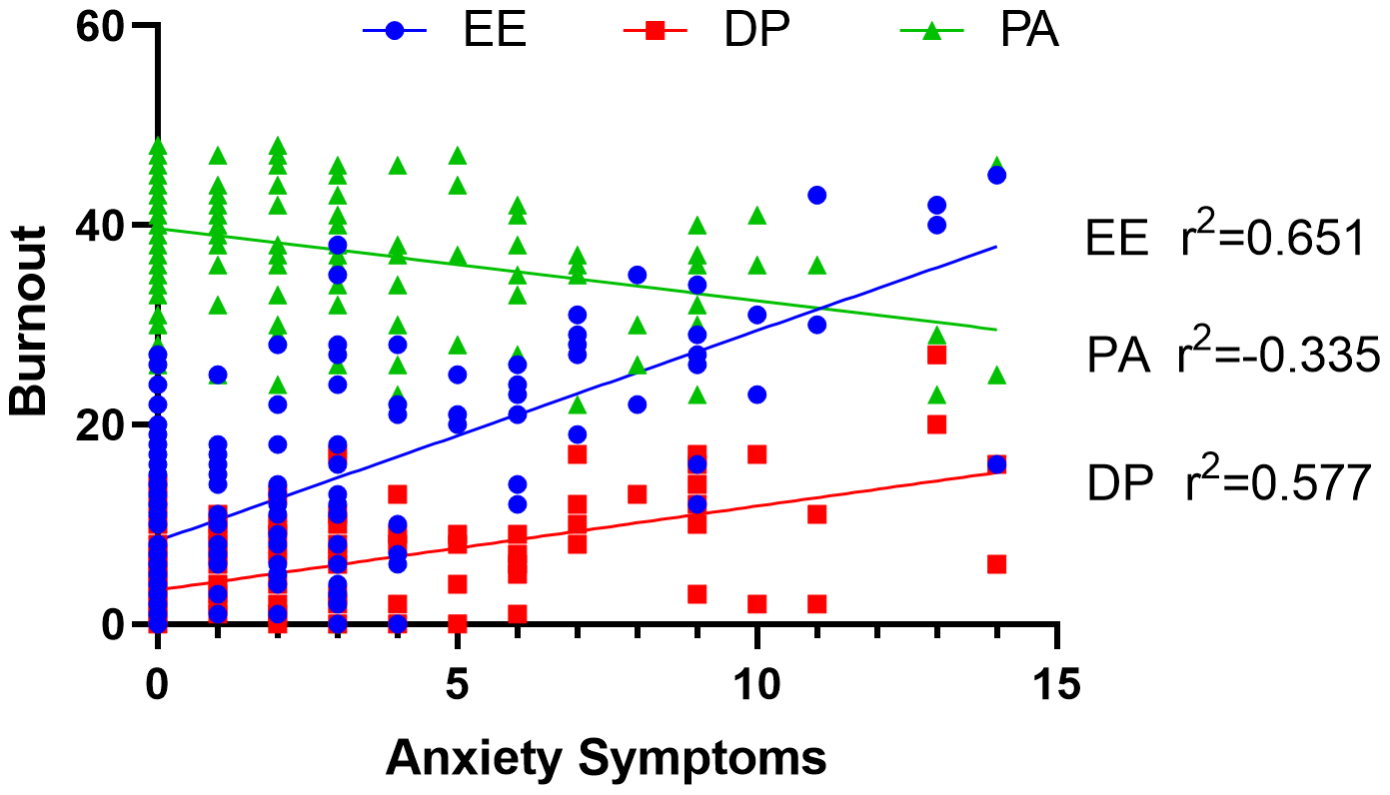
Pearson correlation between anxiety symptoms and burnout.

**Figure 3:**
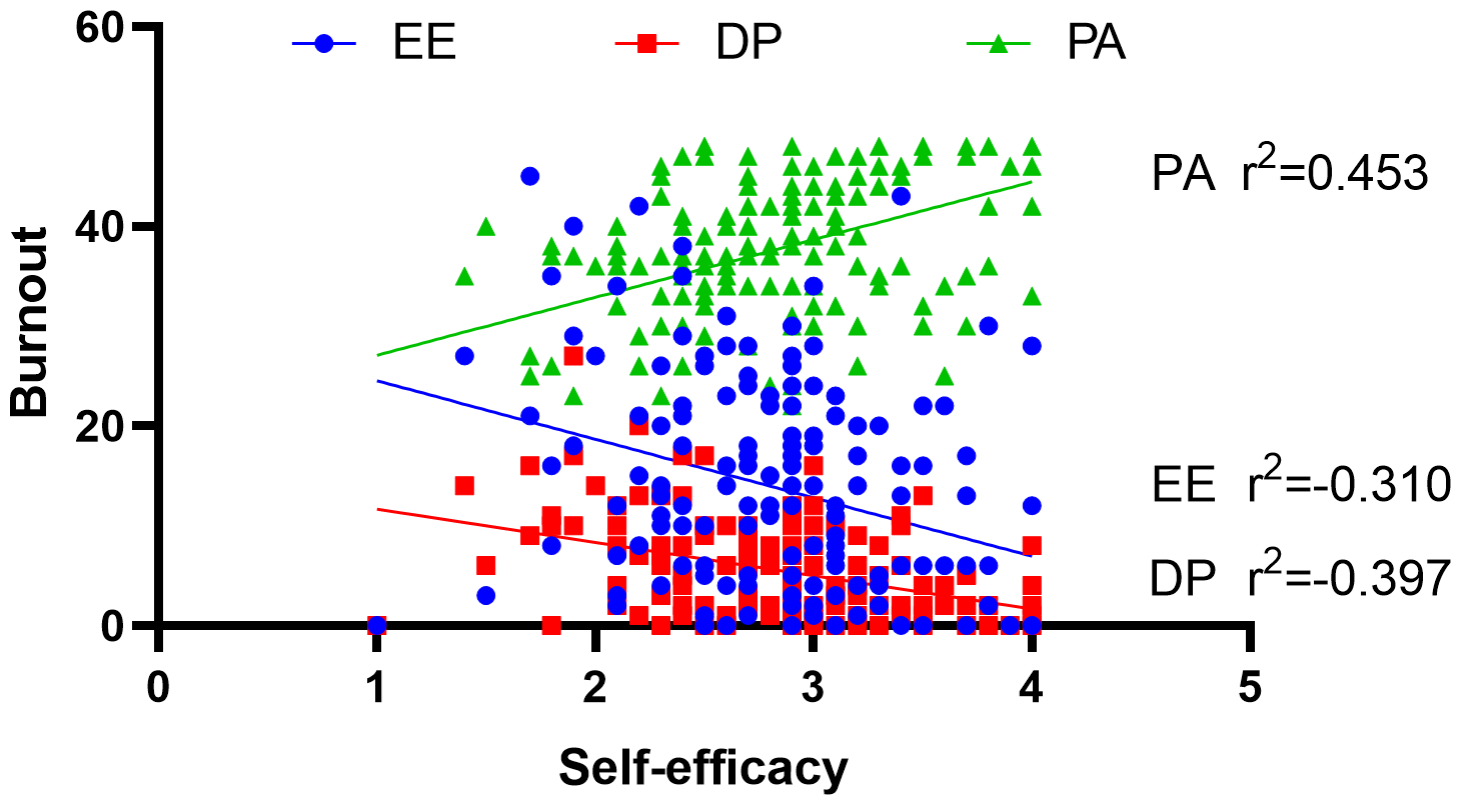
Pearson correlation between self-efficacy and burnout.

Depressive symptoms were positively correlated with age (*r*^2^ = 0.178, *p* = 0.025) and anxiety symptoms (*r*^2^ = 0.164, *p* = 0.039). Anxiety symptoms were positively associated with burnout (*r*^2^ = 0.424, *p* < 0.001) and all three aspects of burnout (Figure 2), depressive symptoms (*r*^2^ = 0.164, *p* = 0.039), and negatively associated with self-efficacy (*r*^2^ = −0.254, *p* = 0.001). Burnout and self-efficacy were negatively correlated (*r*^2^ = −0.312, *p* < 0.001, Figure 3), while both were not correlated with age, sex, or occupation. There was no significant correlation between depressive symptoms and burnout (*r*^2^ = −0.101, *p* = 0.211).

## 4. Discussion

As a global public health crisis, COVID-19 has particularly affected healthcare workers (29). In the early days of the pandemic, many researchers in China focused on the psychological stress of medical personnel and identified several possible contributing factors. Some suggested that during the outbreak, shortages of personal protective equipment (PPE), long working hours, and overwork were common factors affecting the stress levels of healthcare workers (3). In our colleagues’ previous qualitative study (30), FHWs commonly reported nervousness and worrying symptoms, as well as insomnia and physical discomfort. Similar findings were found in several studies (31–34). In a systematic review and meta-analysis of the psychological impact of COVID-19 on Chinese healthcare workers in early 2020 (12), pooled prevalence rates of anxiety, depression, and sleep disturbances were 17% (13–21%), 15% (13–16%), and 15% (7–23%), respectively. Tong et al. (35) reported the prevalence of anxiety and depression in FHWs during the outbreak period and regular period was 1.6 and 13.1% and 6.1 and 8.1%, respectively. In our study, the trend of “less depressed, more anxious” from outbreak to regular period was similar to Tong et al.’s findings, although the prevalence rates of both symptoms were higher in our study. The difference may result from different clinical settings and questionnaires used to evaluate anxiety and depressive symptoms.

Occupational burnout among medical personnel is often higher than in the general population (36). Since the start of the COVID-19 pandemic, studies of burnout among front-line and non-front-line HWs have shown a high burnout rate (20, 37–41), and these HWs often have more emotional distress. Factors related to HWs’ burnout include female sex, less work experience, nurse occupation, and work location (37–41). Using the Effort-Reward Imbalance theory to explore the relationships between burnout and emotional distress, Zhang et al. (42) found that effort and over-commitment were positively associated with depression and anxiety, reward was negatively associated with depression and anxiety, and buffered the harmful effect of effort/over-commitment on depression and anxiety. In our study, FHWs during the outbreak period had lower burnout rates and higher self-efficacy than their colleagues during the regular period. Considering the correlation between burnout and self-efficacy, it is possible that self-efficacy protects FHWs from occupational burnout.

To the general public, COVID-19 has become a constant stressor in the background. One segment of the population that is severely affected by this pandemic is the FHWs. Morioka et al. (43) proposed that some HWs continue to suffer from prolonged psychological distress during the regular period of the COVID-19 pandemic, which may lead to emotional symptoms and somatic discomfort. Risk factors for this include nurse occupation, underlying physical condition, and being prejudiced against due to involvement in COVID-19 healthcare. According to a review of retrospective studies on SARS and MERS (44), an event that occurs over a limited period—however severe—is less traumatic than chronic and prolonged stress over time with no end in sight. Professional identity as a caregiver also makes HWs vulnerable to stress. Yang et al. (45) reported 2,878 out of 15,531 (18.5%) FHWs experienced workplace violence during the outbreak period.

Occupational burnout among HWs is an important issue because it impairs medical quality and safety. Our study revealed the correlation between burnout and anxiety, the latter commonly present under chronic stress, which was found to be correlated with burnout as well (39). Depression and burnout have been considered as synonymous in some literature (46, 47), while others argue that they are categorically distinct (48, 49). We did not find significant correlations between depressive symptoms and burnout in the participants. A recent meta-analysis (48) reported only moderate correlations between scores on burnout and depression measures. One possible explanation for the lack of correlation may be that the tool used to measure burnout in many studies, including this study, is MBI-HSS, which does not include any depressive symptoms (25, 26). Burnout and chronic stress are intertwined and form a vicious circle. Given the increasingly relaxed pandemic control measures and the increasing number of infected patients, FHWs are expected to embrace a wider range and greater intensity of stress. In future studies, follow-up studies on the psychosomatic status and occupational burnout trajectories of FHWs should be continued.

Quoting Dow et al. (50), a crisis – including the COVID-19 pandemic – should never be wasted. Our findings, like others, may provide evidence for tailoring support and intervention plans for FHWs. At the institutional level, there is a need to strengthen the protection and support of FHWs during the pandemic, but proactive prevention against possible psychological distress and occupational burnout should be equally or even more important. The role and function of each individual and the boundaries between roles should be clearly defined (17), and the rotation work pattern should be mandatory to ensure sufficient “off time,” “worry-free time” or “self-care time” for each individual (51). In the routine training of HWs, attention should be paid to reserving personnel in case of special periods such as COVID-19 to be mobilized at any time. At the same time, if there is a shortage of PPEs, priority should be given to ensuring supply at the frontline (9). Psychologically, FHWs should be provided with the necessary psychological support at the individual level, such as counseling and support groups (8, 9, 21). In daily work, enhancing the psychological resilience of HWs also helps to cope with professional burnout (51). Our colleagues have previously reported that in some FHWs, experience at the frontline has led to a more positive assessment of one’s self, and the belief that occupation and life are purposeful and meaningful (30). This may be a sign of good psychological resilience, which affects one’s perception of setbacks (52). It is also important to fully recognize and reward FHWs for their dedication. At the level of government management, short-term responses to the pandemic crisis need to address gaps in the distribution of medical resources in different regions and appropriately increase support for areas lacking medical resources. Since the outbreak of COVID-19, the Chinese Health Commission has organized the transfer of HWs from areas with more medical resources to less developed areas (53). It is important to note that while this measure directly relieved local HW pressures, transferred FHWs faced more complex stressors, such as adaptation needs (54). Long-term measures should focus on further strengthening medical education and enhancing the flexibility and adaptability of HWs.

Our research has some limitations. First, the study was designed to be single-centered, which may limit its generalizability. Second, although we were able to include participants from different periods of the COVID-19 pandemic, we did not follow the same group to see the trend in their emotional distress over time. However, this limitation was compensated for by the fact that the two groups matched in demographic characteristics. Third, we were unable to obtain more sociodemographic information from participants, which may be confounding factors for emotional distress and occupational burnout. Finally, similar to most COVID-19 studies, our study used self-report questionnaires about psychological symptoms rather than diagnostic interviews for mental disorders, which may be affected by recall bias. In future studies, it is recommended to assess factors and coping mechanisms for burnout and psychological symptoms among HWs. It is also important to compare research findings from different cultures and socioeconomic backgrounds.

In conclusion, anxiety, depressive symptoms and burnout are prevalent in FHWs during both the outbreak period and the regular period of COVID-19. There is a tendency to be less depressed, but more anxious and burned out over time, although the severity of the pandemic is decreasing. Self-efficacy may be an important factor in protecting FHWs from occupational burnout. During the regular period of COVID-19, more attention and active interventions are still needed for the mental health and occupational burnout of healthcare workers.

## Data availability statement

The raw data supporting the conclusions of this article will be made available by the authors, without undue reservation.

## Ethics statement

The studies involving human participants were reviewed and approved by the Ethics Committee of Peking Union Medical College Hospital. Written informed consent for participation was not required for this study in accordance with the national legislation and the institutional requirements.

## Author contributions

WG, JC, XH, and JW conceived and designed the study. WG, JJ, JH, and YD collected the data. WG performed the statistical analyses. WG and JC wrote the first draft of the manuscript draft. All authors had access to the data, played a role in writing the manuscript, and commented on the posterior versions and approved the final manuscript.

## Funding

This study was supported by the Capital Funds for Health Improvement and Research (project number: CFH 2022–2-4012) and the STI2030-Major Projects (project number: 2021ZD0202001).

## Conflict of interest

The authors declare that the research was conducted in the absence of any commercial or financial relationships that could be construed as a potential conflict of interest.

## Publisher’s note

All claims expressed in this article are solely those of the authors and do not necessarily represent those of their affiliated organizations, or those of the publisher, the editors and the reviewers. Any product that may be evaluated in this article, or claim that may be made by its manufacturer, is not guaranteed or endorsed by the publisher.
